# Hurst-Kolmogorov Process is a More Reliable and Statistically Powerful Alternative to Detrended Fluctuation Analysis for Estimating Hurst in Short Walking Trials

**DOI:** 10.1007/s10439-026-04011-1

**Published:** 2026-02-13

**Authors:** Vasileios Mylonas, Tyler M. Wiles, Seung Kyeom Kim, Nick Stergiou, Aaron D. Likens

**Affiliations:** 1 Department of Biomechanics, University of Nebraska at Omaha, 6001 Dodge St, Omaha, Nebraska 68182 USA; 2 Department of Physical Education and Sport Science, Aristotle University of Thessaloniki, Thessaloniki, Greece

**Keywords:** Gait variability, Intraclass correlation coefficient, Power analysis, Fractal analysis, Nonlinear analysis

## Abstract

**Background:**

For decades, researchers have used Detrended Fluctuation Analysis (DFA) as a method to assess the temporal structure of gait variability through the Hurst exponent (*H)*. However, DFA’s reliance on long time series limits reliability and reduces statistical power when applied to short walking trials, restricting its applicability. The Hurst-Kolmogorov process (HKp), an increasingly common algorithm, may offer more reliable and efficient estimates of the *H* in short walking trials. This study evaluated the reliability and statistical power of HKp versus DFA in estimating *H* from gait kinematics using short time series.

**Methods:**

119 healthy adults (34 young, 57 middle-aged, and 38 older) were sampled from the NONAN GaitPrint dataset. Each participant walked 9 four-minute trials per day over the course of two days, which were a week apart. *H* was estimated for stride interval, stride length, and the lower limb joint range of motion using DFA and HKp. Kinematic variables were calculated for time series ranging from 50 to 175 strides. Intraclass correlation coefficients (ICCs) were calculated between days using the average of 1 to 9 trials per day. Power estimations using simulated time series were performed to assess the ability of each method to detect group differences under varying effect sizes, sample sizes, trial numbers, and time series lengths.

**Results:**

HKp achieved excellent reliability (ICC > 0.90) in short trials (<100 strides), whereas DFA rarely exceeded moderate reliability. Statistical power simulations demonstrated that HKp yielded higher power than DFA, particularly when fewer trials and subjects were available. A summary table is provided to guide sample size selection under different design conditions.

**Conclusion:**

HKp offers a more reliable and statistically powerful alternative to DFA for estimating *H* in short walking trials. These findings support HKp as a practical tool for assessing the temporal structure of gait variability, improving feasibility in experimental and research settings.

## Introduction

Walking is a repetitive activity where each step follows another step, yet consecutive steps are never identical. Instead, each step differs slightly from the next in gait features such as the duration and length of each step. Analytical approaches that can capture the temporal sequence of these variations, known as nonlinear analyses, reveal that variations in consecutive steps are correlated [1–6]. In healthy gait, the correlation among a series of steps demonstrates strong *persistence*, where faster strides tend to follow faster strides, while slower strides tend to follow slower ones. Moreover, gait variability exhibits a specific structure across time scales: strides that are closer in time exhibit a stronger correlation that gradually decays as their temporal separation increases. This specific temporal structure that has been termed as “optimal” is commonly observed in young healthy adults, whereas older adults and patients often exhibit reduced correlations between strides [5, 7–11].

**Fig. 1:**
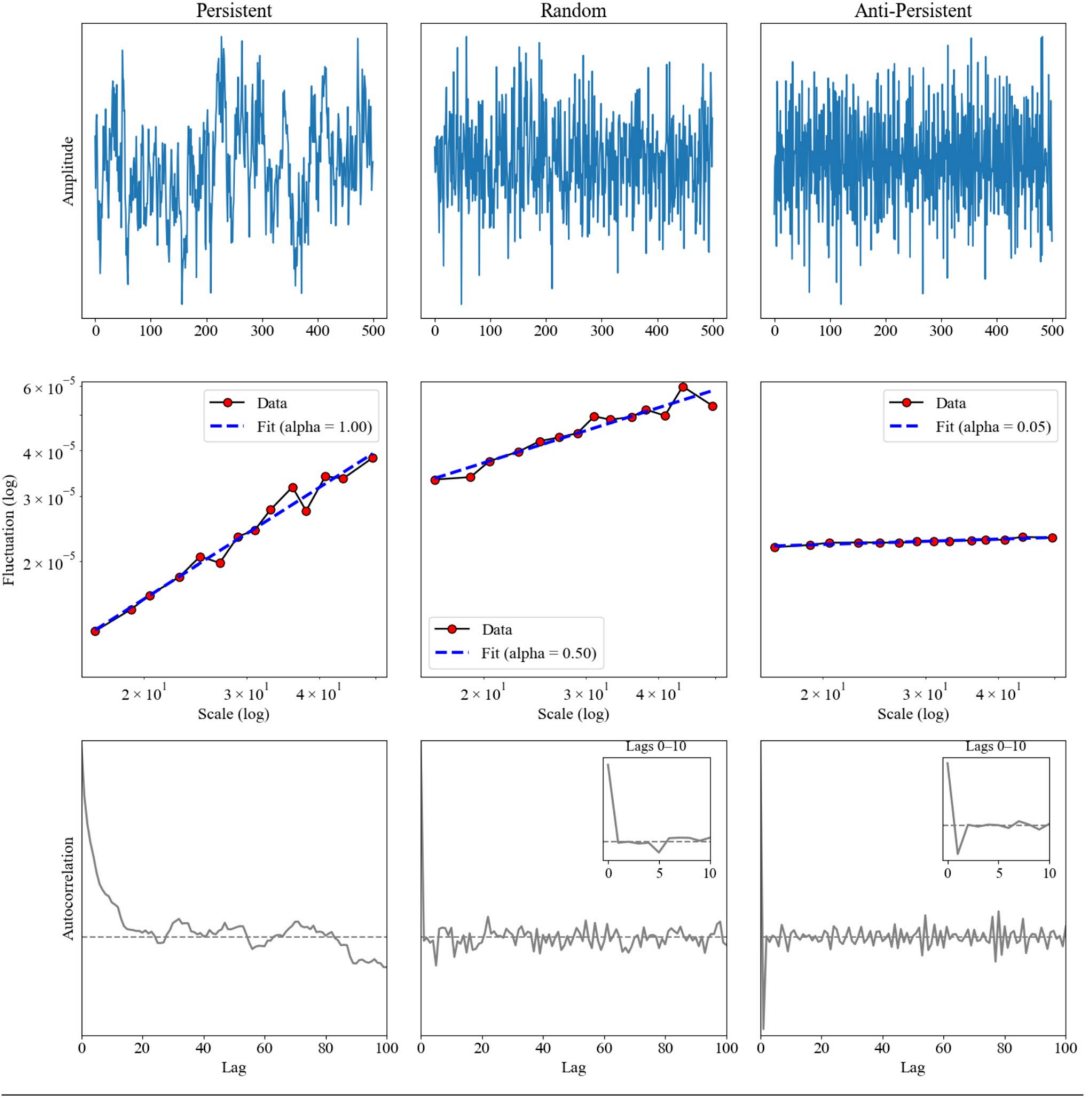
Representative time series (top row), DFA fluctuation plots (middle row), and autocorrelation functions (bottom row) for persistent (left), random (middle), and anti-persistent (right). The DFA scaling exponent α is derived from the slope of the linear fit on the log–log fluctuation plot and equals the Hurst exponent H only when the signal is stationary. Persistent signals (*α* > 0.5) show slowly decaying autocorrelations; uncorrelated signals (*α* ≈ 0.5) exhibit flat autocorrelation; anti-persistent signals (*α* < 0.5) exhibit negative short-lag autocorrelations. Autocorrelation quantifis how the time series values are related to one another across increasing time separations. In this chase of stationary signals, α approximates the Hurst exponent. The autocorrelation function of fractional Gaussian noise is defined as:$\rho_{k} = \frac{1}{2}\left| { k + 1} \right|^{2H} + \frac{1}{2}\left| { k - 1} \right|^{2H} - \left| k \right|^{2H} , k = 0,1, \ldots ,K$ where *H* is the Hurst exponent and *ρ*ₖ is the autocorrelation at lag k up to a maximum lag, *K*. Inset panels (bottom row) provide a zoomed view of short-lag autocorrelation (lags 0–10)

Detrended fluctuation analysis (DFA) is one of the most widely used nonlinear methods to examine the persistence of time series, such as stride intervals [12–18]. The outcome of DFA is the scaling exponent *α* (alpha), which characterizes how the correlations between stride intervals change with increasing time separation (Figure 1). When the underlying time series is stationary (*α* < 1.0), *α* is equivalent to the so-called Hurst exponent (*H*). An *H* of 0.5 indicates the absence of temporal correlations (i.e., a random or uncorrelated series). Values of *H* between 0.5 and 1.0 reflect statistically persistent behavior (large values are likely followed by large values, and small values are likely followed by small values). *H* values between 0 and 0.5 indicate anti-persistent behavior (small values are likely followed by large values and vice versa).

Nonlinear analysis methods such as DFA have not gained widespread adoption in clinical settings, despite being widely used in research. Reaching a consensus on the practical implementation of these tools is a critical step toward their clinical integration. A key limitation of DFA is its reliance on large time series (typically more than 512 strides) to produce meaningful and reliable *H* estimates [12, 13, 19, 20]. Collecting such a large number of strides often requires over 10 minutes of continuous walking, a probable and considerable challenge for older adults with mobility deficits and many other clinical groups [14, 19, 21]. Attempts to overcome this limitation by 'stitching' together short gait trials have been shown to produce unreliable scaling exponents [22]. Additionally, even with sufficiently long trials, DFA exhibits systematic bias at both low and high ends of the spectrum (*H* approaching 0 or 1). Finally, DFA is sensitive to time series length, even for time series that exceed 512 data points, thereby limiting the validity of comparisons between trials of differing durations [13].

Due to these algorithmic shortcomings, DFA estimates of *H* often lack reliability, further limiting their applicability [14, 23–26]. Reliability, as quantified by intraclass correlation (ICCs), reflects the degree of correlation and agreement among repeated measurements [27, 28]. Values below 0.5 indicate poor reliability, values between 0.5 and 0.75 suggest moderate reliability, values between 0.75 and 0.9 are generally considered good, and values exceeding 0.9 are considered excellent [27, 28]. When using DFA with gait data, *H* estimates produce poor reliability for short trials (around three minutes) and only moderate to good reliability for longer trials exceeding eight minutes [14]. When multiple trials are collected within a day and *H* is averaged across trials, between-day reliability improves, with ICCs often exceeding 0.85 [23, 24]. However, reliability is only moderate when the multiple trials used are short (less than 5 minutes of walking) [24].

Those reliability findings primarily stem from analyses of spatiotemporal time series. To that point, research in that area shows that ICC for stride interval *H* estimates (0.870) tends to be higher than for stride length (0.722) [23]. Reliability of that magnitude is certainly good enough for research purposes, but the exclusive focus on stride intervals and lengths leaves considerable uncertainty about the utility of DFA in other settings and with other variables of interest. For example, our team has recently calculated *H* for kinematic variables, such as joint range of motion, and found differences between young and older adults, particularly in hip ROM [11]. Other research groups have utilized DFA to characterize ground reaction forces [29, 30] and electromyographic activity [17, 31] generated during walking, postural sway structure during quiet standing [32–35], and the structure of heart rate variability [36–39]. The growing interest to apply DFA in other settings indicates that it is necessary to study the reliability of *H* in variables such as those just mentioned–work that remains unexplored. Additionally, it is unclear whether reliability patterns are consistent across age groups.

Beyond reliability concerns, the increased error variance associated with the DFA algorithm may also undermine its downstream statistical power. This elevated variance may obscure true differences between groups by increasing within-group variability attributable to algorithmic error. Thus, an increased Type II statistical error would result from failing to reject a false null hypothesis. Prior work has demonstrated that the statistical power of DFA estimates in short time series (< 512 strides) often falls below acceptable thresholds (< 0.8) but can be improved with larger sample sizes and an increased number of trials per subject [40]. However, those results are based on a single effect size corresponding to a mean difference of 0.1 in *H*, which may overestimate the magnitude of group differences typically observed in experimental settings [5, 20, 41]. Algorithmically induced reduction in statistical power can further hinder the application of DFA and, consequently, the clinical integration of metrics that characterize the temporal structure of variability. Moreover, the minimum sample size required to reach conventional power thresholds across different effect sizes, trial counts, and algorithms has not been established.

Unlike DFA, a more recently developed algorithm, the Hurst-Kolmogorov process (HKp) provides accurate *H* estimates with as few as 64 data points [13, 40, 42, 43]. Full descriptions appear in the methods section, but we briefly distinguish the two methods here. Unlike DFA, which quantifies how the fluctuations increase across time scales, the HKp approach uses the theoretical autocorrelation structure expected from an HKp time series and applies a Bayesian sampling method to estimate a distribution of the most likely *H* [43, 44]. Despite these methodological differences, both algorithms estimate *H* for stationary time series [13, 42, 45]. Both numerical simulations and empirical data have shown that HKp outperforms DFA, particularly in short time series, by more accurately estimating *H* [13, 42]. Additionally, HKp exhibits minimal dispersion around its central tendency, implying reduced error variance compared to DFA. Unlike DFA, those properties remain unaffected by either the underlying correlation structure or the length of the time series [13, 42]. As such, HKp represents a promising alternative to DFA, potentially overcoming key limitations such as poor reliability and reduced statistical power in short recordings. However, whether the improved estimation accuracy and minimal dispersion associated with HKp meaningfully translate in between day reliability and reduced sample size requirements to achieve conventional thresholds of statistical power have not been systematically evaluated. Given those advantages, HKp could emerge as a practical nonlinear tool for estimating *H* across a wider range of clinical and behavioral applications.

This study aims to evaluate the performance of HKp and DFA in estimating *H* from various kinematic variables during short walking trials. Specifically, we assess the day-to-day reliability of *H* estimates for stride interval, stride length, and joint range of motion across young, middle-aged, and older adults using trials composed of 50–175 strides. Additionally, our second aim applies both algorithms to simulated time series to evaluate their statistical power in detecting differences between groups across a range of effect sizes, trial counts, and sample sizes. To support future study design, we also report the minimum number of participants required to achieve 80% power under each condition. We hypothesize that HKp will produce more reliable estimates than DFA, with reliability improving as a function of the number of strides and trials. Furthermore, we expect HKp to produce greater statistical power compared to DFA, with power increasing as a function of the number of trials and subjects per group.

## Methods

### Empirical Experiment

#### Empirical Data Description

119 participants were sampled from the NONAN GaitPrint dataset [11, 46, 47]. This dataset comprises three groups of healthy young, middle-aged, and older adults (Table 1). The original data collection protocols were approved by the Institutional Review Board, and all participants provided written informed consent prior to participation, as detailed in the original dataset publications. The present study involved only secondary analysis of fully anonymized data and did not require additional ethical approval. Full-body kinematic data were collected using a full body Inertial Measurement Unit (IMU) motion capture system (Noraxon Ultium Motion^TM^; Noraxon, Inc., Scottsdale, AZ), recording at 200 Hz. Raw data were recorded using the MyoResearch software (version 3.18.126; Noraxon USA Inc., Scottsdale, AZ). Participants completed 18 four-minute trials over the course of two days, spaced one week apart (9 per day). Standardized procedures were implemented to minimize IMU drift, including a functional calibration performed prior to each trial. Detailed information regarding calibration procedures, and drift-mitigation strategies is provided in the original dataset publications. All data collections were performed overground on an indoor track. Participants of the present study met the following criteria: (i) ability to provide informed consent; (ii) ability to walk independently without the use of assistive devices; (iii) no self-reported diagnosis of neurological disorders; and (iv) no self-reported history of lower limb disability, injury, or disease.

**Table 1:** Means and standard deviations of age, height, and weight by group. M and SD represent mean and standard deviation, respectively

Group	Female (n)	Male (n)	Age (M ± SD)	Height (M ± SD)	Weight (M ± SD)
Young	16	18	24.44 ± 2.57	173.81 ± 7.87	72.13 ± 15.15
Middle	26	21	46.00 ± 6.03	173.28 ± 8.54	85.95 ± 17.54
Older	19	19	64.61 ± 6.94	171.33 ± 9.96	79.77 ± 16.50

#### Data Analysis

Gait events (heel strikes) and lower-limb joint kinematics were derived within MyoResearch using a biomechanical model. Segment orientations were estimated using the IMU signals, and joint angles were computed from the relative orientations of adjacent segments. Anthropometric measurements were used to scale the biomechanical model and reconstruct subject-specific joint kinematics. We used the HKp and DFA algorithms to calculate *H*. *H* estimates were derived from spatiotemporal variables, including stride interval and stride length, and from kinematic variables, including joint ranges of motion (ROM) for the hip, knee, and ankle in the sagittal plane. For each variable, time series were extracted from individual walking trials and processed separately.

We used DFA to calculate *H* for the stride interval and the simulated time series [15]. For DFA to estimate *H*, the time series *x*_*t*_ was integrated and divided into windows of size *n*. The data points within each window were then fitted with an ordinary least squares regression; the integrated data were then detrended by calculating the residuals. The mean squared residual was calculated within each window of size *n*. Then, the average was computed across all windows of size *n,* the square root of which defined the fluctuation function, *F(n)*. This process was repeated across multiple window sizes. After iterating through all window sizes, log(F(*n*)) was plotted against log(*n*), and a linear regression was performed. The slope of this linear regression line represents the Hurst exponent. The range of time scales (window sizes) selected for this analysis was 4 to *N*/4. This range was selected to provide a wide range of window sizes for short time series, as commonly used alternative ranges (e.g., 16 to N/9) [12] are not applicable for very short stride series. DFA was performed using the *dfa()* function from the *fractalRegression* R package [48].

The *H* of the stride interval and simulated time series was also calculated using the HKp [13, 42, 43]. The HKp utilizes a Bayesian accept-reject algorithm to estimate *H*. Posterior distributions of H, f(x), are sampled based on the autocorrelation function for the Hurst-Kolmogorov process, which is given by

$$\rho_{k} = \frac{1}{2}\left| { k + 1} \right|^{2H} + \frac{1}{2}\left| { k - 1} \right|^{2H} - \left| k \right|^{2H} , k = 0,1, \ldots ,$$

where $k$ is the time lag and $\rho_{k}$ is the autocorrelation for a given time lag, $k$. Then, the accept-reject algorithm is used to sample $H$. First, candidate values of *H* are sampled from a uniform distribution ranging from 0 to 1. Second, the posterior density at the candidate value, $f\left( {H_{cand} } \right)$, is evaluated. Next, a second random number, u, is drawn from a uniform distribution ranging from 0 to 1. The candidate is accepted if the u is no larger than $f\left( {H_{cand} } \right)$ divided by the peak density of $f\left( x \right)$. The peak density of $f\left( x \right)$ is found using numerical optimization. Otherwise, the candidate is rejected. This procedure was iterated until 50 H values were sampled, to which the median of the 50 sampled *H* values was taken as the point estimate of *H*. Previous simulation work has shown that this number of samples is adequate to provide an accurate *H* estimate [42]. For a detailed description of the posterior distribution sampling and the accept-reject algorithm, we refer the readers to its original citation [13].

#### Statistical Analysis

Reliability was assessed using the Intraclass Correlation Coefficient (ICC), computed using the *ICC()* function of the *psych* R package [49]. A two-way mixed-effects model with consistency agreement (ICC [3, k]) was used for all comparisons [27]. ICCs were calculated between days using the average of 1 to 9 trials per day. This analysis was repeated for *H* estimates derived from time series ranging in length, consecutively, from 50 to 173 strides. For all time series lengths, consecutive strides were selected from the end of each trial rather than the beginning to minimize potential familiarization effects at trial onset. The lower bound was selected to allow investigation of adequately short time series, while the upper bound reflects the maximum number of strides completed by all participants. This analysis was repeated separately for each age group to evaluate the Hurst exponent’s reliability across the lifespan.

### Simulation Experiment

#### Simulated Data Description

Given the increased error variance in *H* that is expected when derived from DFA, we complemented our empirical reliability analysis with a simulation experiment to examine the statistical power of detecting true differences between groups. This approach allowed us to systematically quantify how study design factors such as sample size, trial count, and time series length affect the ability to detect between-group differences in *H*. For the second aim of the study, we performed statistical simulations to estimate the observed power in a between-group, unpaired t-test design. To enhance the ecological validity of our simulations, we generated four trials per simulated subject with an intraclass correlation coefficient (ICC) of 0.65, consistent with the between-trial reliability observed in the empirical data’s results. To reproduce this correlation structure in our simulated dataset, we employed Cholesky decomposition, a matrix factorization technique that allows for the generation of multivariate normal data with a specified covariance matrix [50]. Using this technique, we constructed a covariance matrix that yielded the desired ICC of 0.65 among the simulated trials. Each simulated group consisted of 10 to 80 subjects. To ensure adequate power estimation for small mean differences (*Δ*H = 0.04), we simulated up to 250 subjects per group because the conventional sample range (up to 80 per group) failed to reach the 0.80 power threshold. For *Δ*H values of 0.08 and 0.12, simulations were performed using 80 subjects per group, as these conditions consistently achieved sufficient power within that range. Simulated time series were generated using the *fGn_sim()* function from the *fractalRegression* package in R [48]. Simulated time series were created at multiple lengths (50, 100, 150, and 200 strides) to assess how time series length influences power.

Each simulated time series was analyzed using both DFA and HKp algorithms to estimate *H*. Three simulations were performed, each representing a different between-group effect size by adjusting the mean *H* of Group B. The mean *H* for Group A was fixed at 0.75 in all simulations, while the difference between the two groups (*Δ*H) was 0.04, 0.08, and 0.12 for each simulation, respectively. Within-group standard deviations were set at 0.11 for both groups to reflect the empirical variability observed in stride interval Hurst estimates. No additional variance was introduced between trials within each group; all trials were simulated with the same numerical mean (0.75) and standard deviation (0.11). To evaluate how the number of subjects and trials per subject affected the observed power, we repeated simulations across varying sample sizes (10 to 80 subjects per group) and trial counts (1 to 4 trials per subject). When multiple trials were simulated, *H* was averaged across trials for each subject, and the mean *H* was used to evaluate the statistical power of detecting the between group difference. We performed 1,000 simulations per condition to estimate statistical power for a between-subjects t-test at α = 0.05. A summary of all simulation parameters and design conditions is provided in Table 2.

**Table 2:** Simulation parameters used to estimate statistical power for detecting between-group differences in H

Parameter	Values Used	Purpose
Number of subjects per group	10 to 250 (*ΔH* = 0.04), 10 to 80 (*ΔH* = 0.08, 0.12)	To assess how sample size affects power and ensure sufficient power estimation for small effect sizes
Number of trials per subject	1 to 4	To assess how averaging across trials affects power
ICC between trials	0.65	Reflects empirical between-trial reliability
Time series length	50, 100, 150, 200	To assess how time series length affects power
Effect sizes (*ΔH*)	0.04, 0.08, 0.12	Small, moderate, and large between-group differences
Within-group SD	0.11	Based on empirical variability in stride interval *H*
Simulation repetitions	1000 per condition	To estimate power reliably

Both the Hurst-Kolmogorov process (HKp) and Detrended Fluctuation Analysis (DFA) were applied to each simulated time series to compare their power across conditions

#### Statistical Analysis

The dependent measure was the average *H* across trials. Statistical power was estimated as the proportion of tests out of 1000 in which the null hypothesis was rejected at the *α* = 0.05 level. All simulations and analyses were implemented using the R programming language [51].

## Results

### Human Subjects: Reliability of Hurst Exponent Estimates

**Fig. 2:**
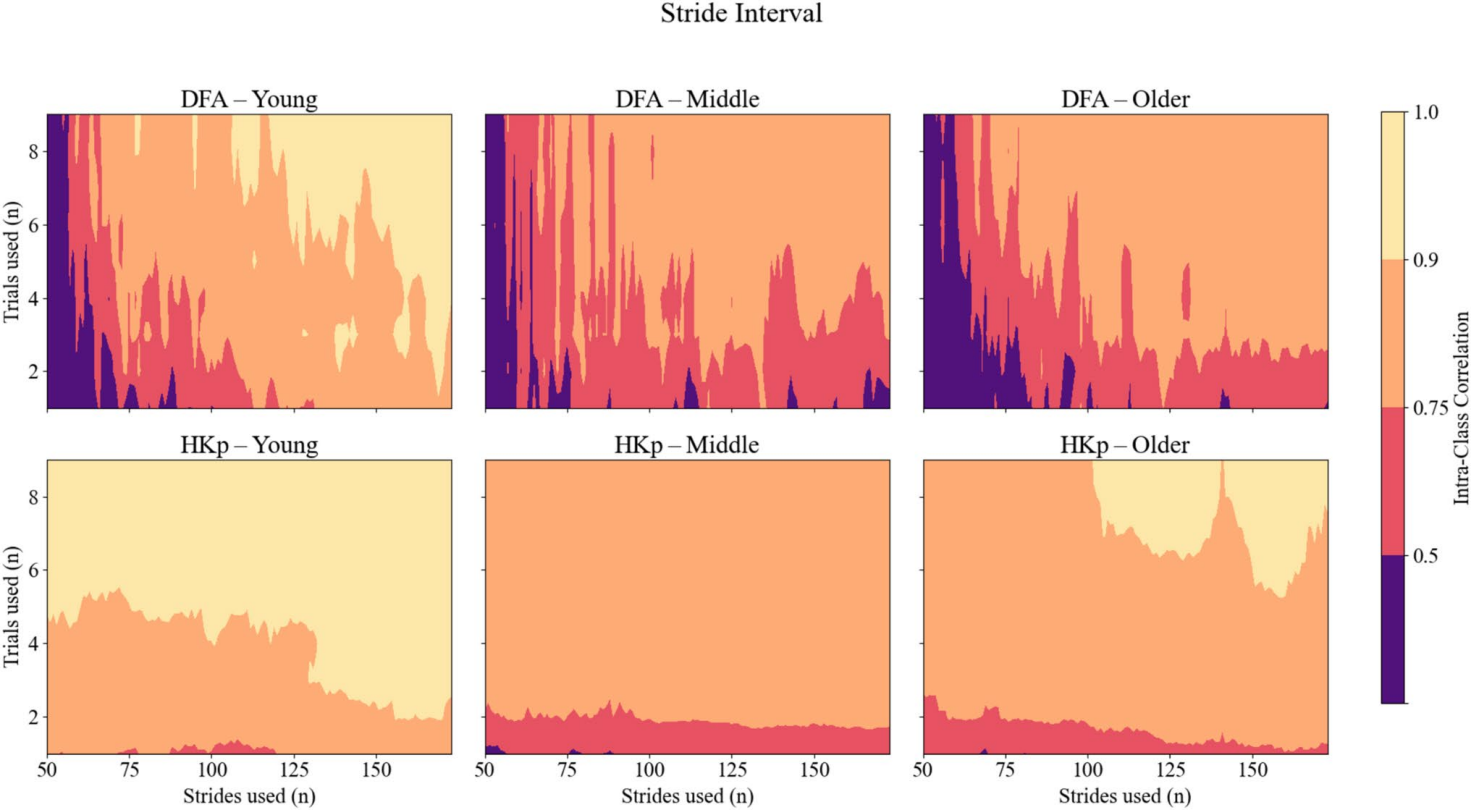
Intra-class correlation coefficients (ICC) representing between-day reliability of *H* estimates for stride interval time series across three age groups: young (left column), middle-aged (center column), and older adults (right column). Reliability is shown for two algorithms—Detrended Fluctuation Analysis (DFA; top row) and Hurst-Kolmogorov process (HKp; bottom row). The x-axis represents the number of strides per trial; the y-axis represents the number of trials used. Color gradients indicate reliability levels: purple (poor, ICC < 0.50), red (moderate, 0.50–0.75), orange (good, 0.75–0.90), and yellow (excellent, ICC > 0.90)

We first evaluated between-day reliability for each algorithm using empirical gait data stratified by age group. Between-day reliability of *H* estimates for stride interval and stride length varied across stride counts and trial numbers in young, middle-aged, and older adults. DFA yielded generally poor to moderate reliability, especially with fewer and shorter trials (Figure 2). In contrast, HKp consistently produced higher ICC values across all conditions. For stride intervals (Figure 2), DFA performance was unstable and unreliable across conditions, with ICC values frequently falling below 0.50, especially for short time series. HKp achieved good to excellent reliability (ICC > 0.75 and > 0.90, respectively) with as few as three trials and 100 strides. Even at lower stride counts, HKp maintained relatively high reliability when more trials were included.

**Fig. 3:**
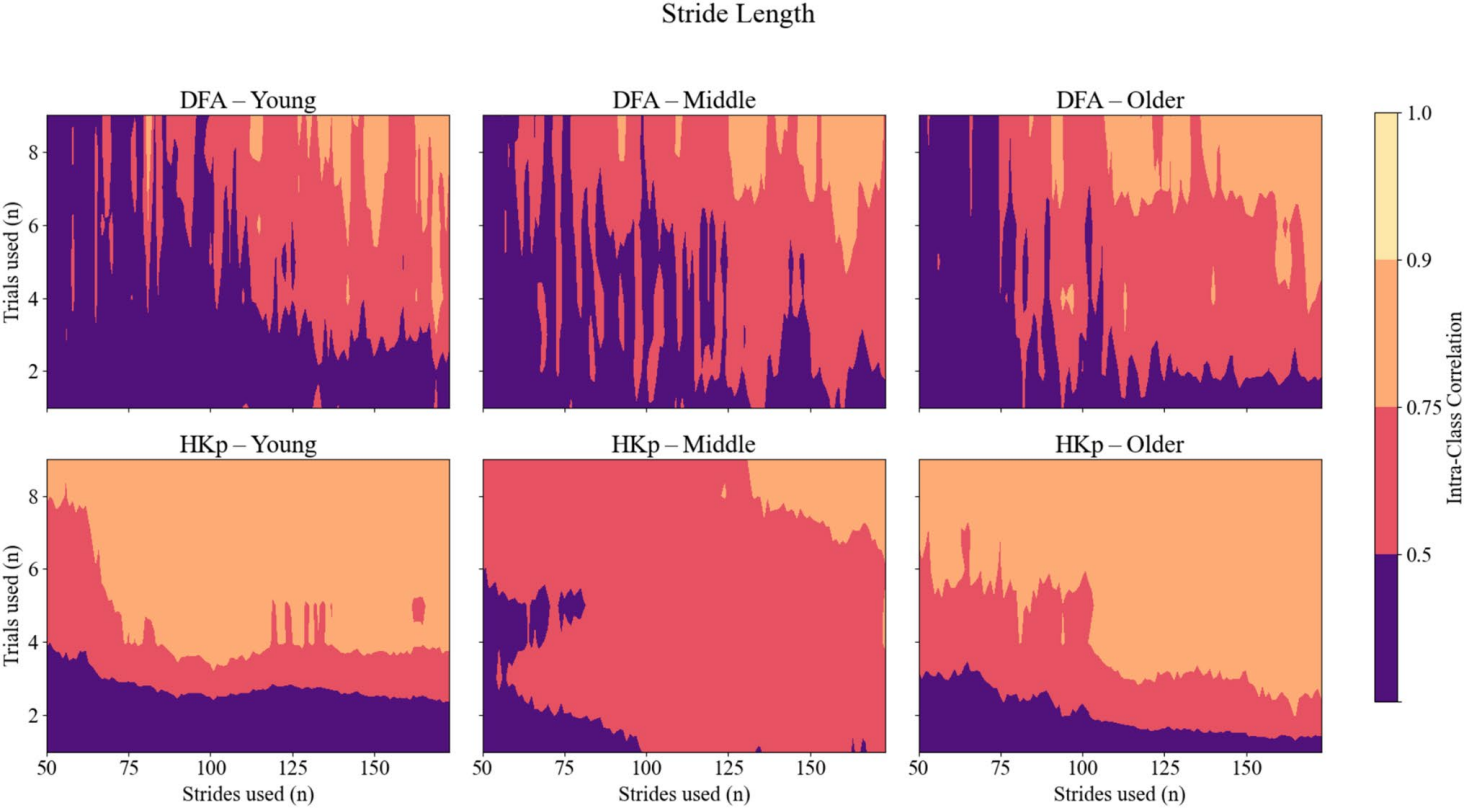
Intra-class correlation coefficients (ICCs) representing between-day reliability of *H* estimates for stride length time series across three age groups: young (left column), middle-aged (center column), and older adults (right column). Reliability is shown for two algorithms—Detrended Fluctuation Analysis (DFA; top row) and Hurst-Kolmogorov process (HKp; bottom row). The x-axis represents the number of strides per trial; the y-axis represents the number of trials used. Color gradients indicate reliability levels: purple (poor, ICC < 0.50), red (moderate, 0.50–0.75), orange (good, 0.75–0.90), and yellow (excellent, ICC > 0.90)

A similar pattern was observed for stride lengths (Figure 3). DFA produced consistently poor to moderate reliability across all age groups even with an increased number of strides and trials used. On the contrary, HKp showed improved ICCs, though overall ICCs for stride length were lower than for stride interval. Among the three groups, older adults exhibited the most consistent benefit from HKp, reaching good ICCs with fewer strides and trials used.

**Fig. 4:**
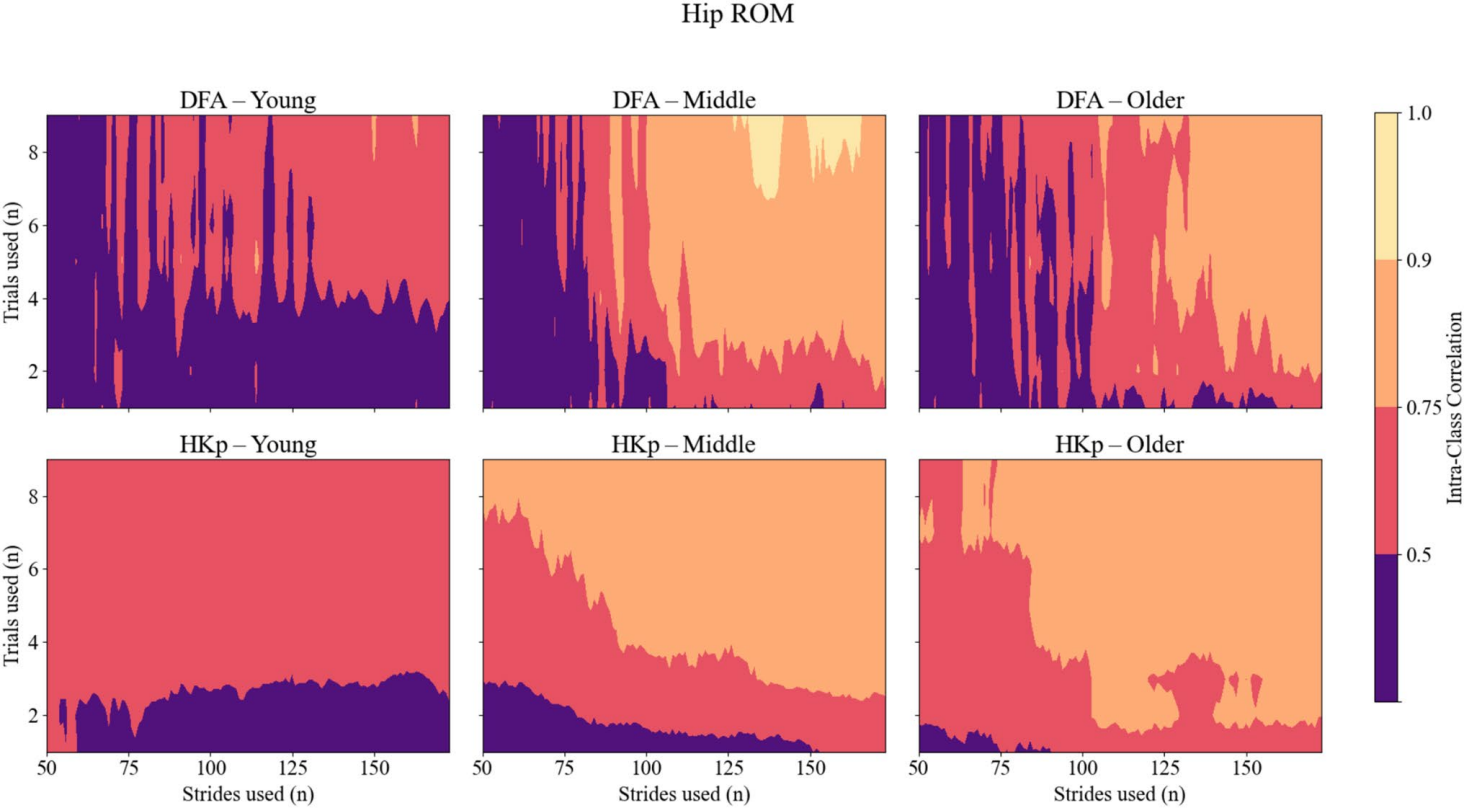
Intra-class correlation coefficients (ICCs) representing between-day reliability of *H* estimates for Hip joint ROM time series across three age groups: young (left column), middle-aged (center column), and older adults (right column). Reliability is shown for two algorithms—Detrended Fluctuation Analysis (DFA; top row) and Hurst-Kolmogorov process (HKp; bottom row). The x-axis represents the number of strides per trial; the y-axis represents the number of trials used. Color gradients indicate reliability levels: purple (poor, ICC < 0.50), red (moderate, 0.50–0.75), orange (good, 0.75–0.90), and yellow (excellent, ICC > 0.90)

**Fig. 5:**
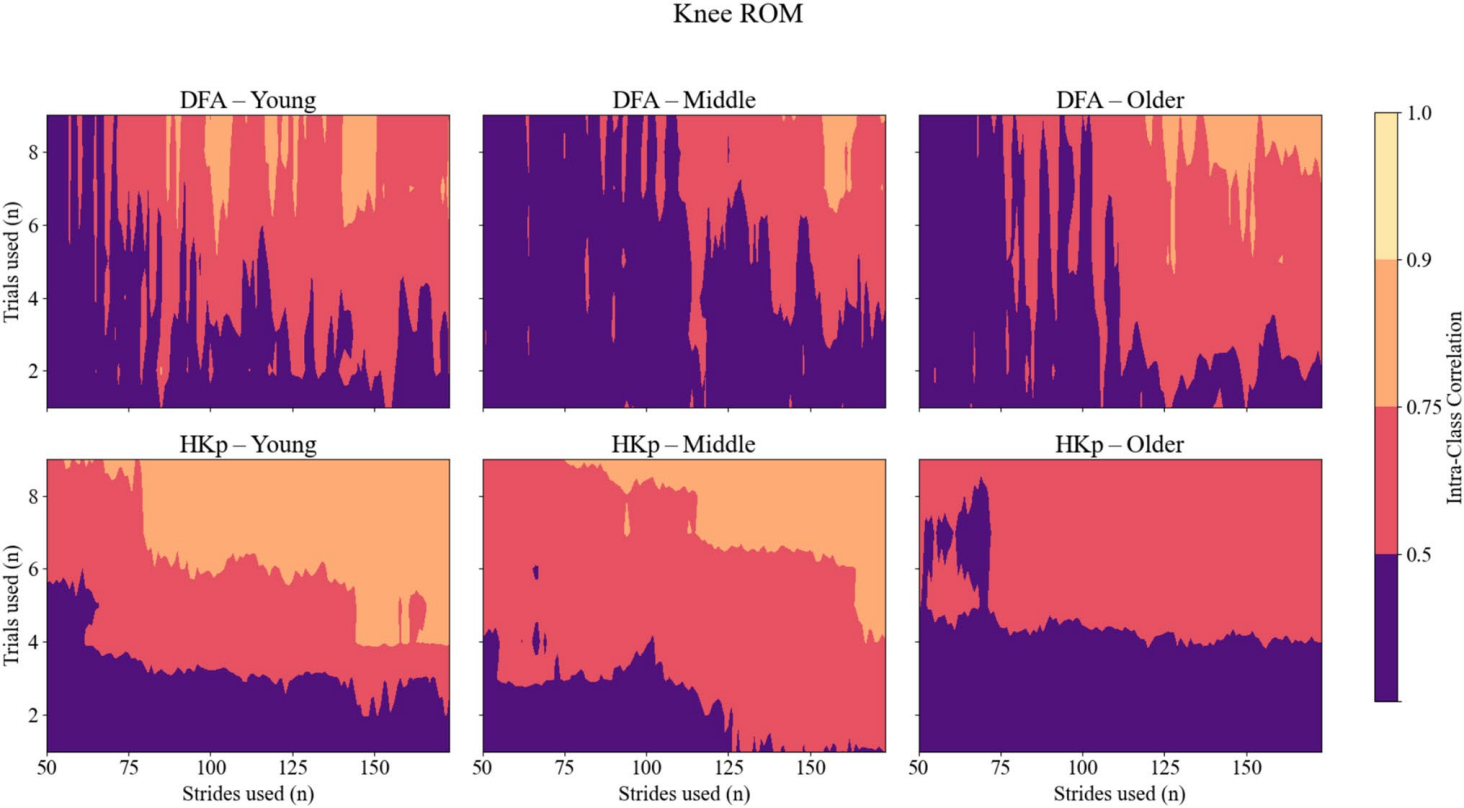
Intra-class correlation coefficients (ICCs) representing between-day reliability of *H* estimates for Knee joint ROM time series across three age groups: young (left column), middle-aged (center column), and older adults (right column). Reliability is shown for two algorithms—Detrended Fluctuation Analysis (DFA; top row) and Hurst-Kolmogorov process (HKp; bottom row). The x-axis represents the number of strides per trial; the y-axis represents the number of trials used. Color gradients indicate reliability levels: purple (poor, ICC < 0.50), red (moderate, 0.50–0.75), orange (good, 0.75–0.90), and yellow (excellent, ICC > 0.90)

**Fig. 6:**
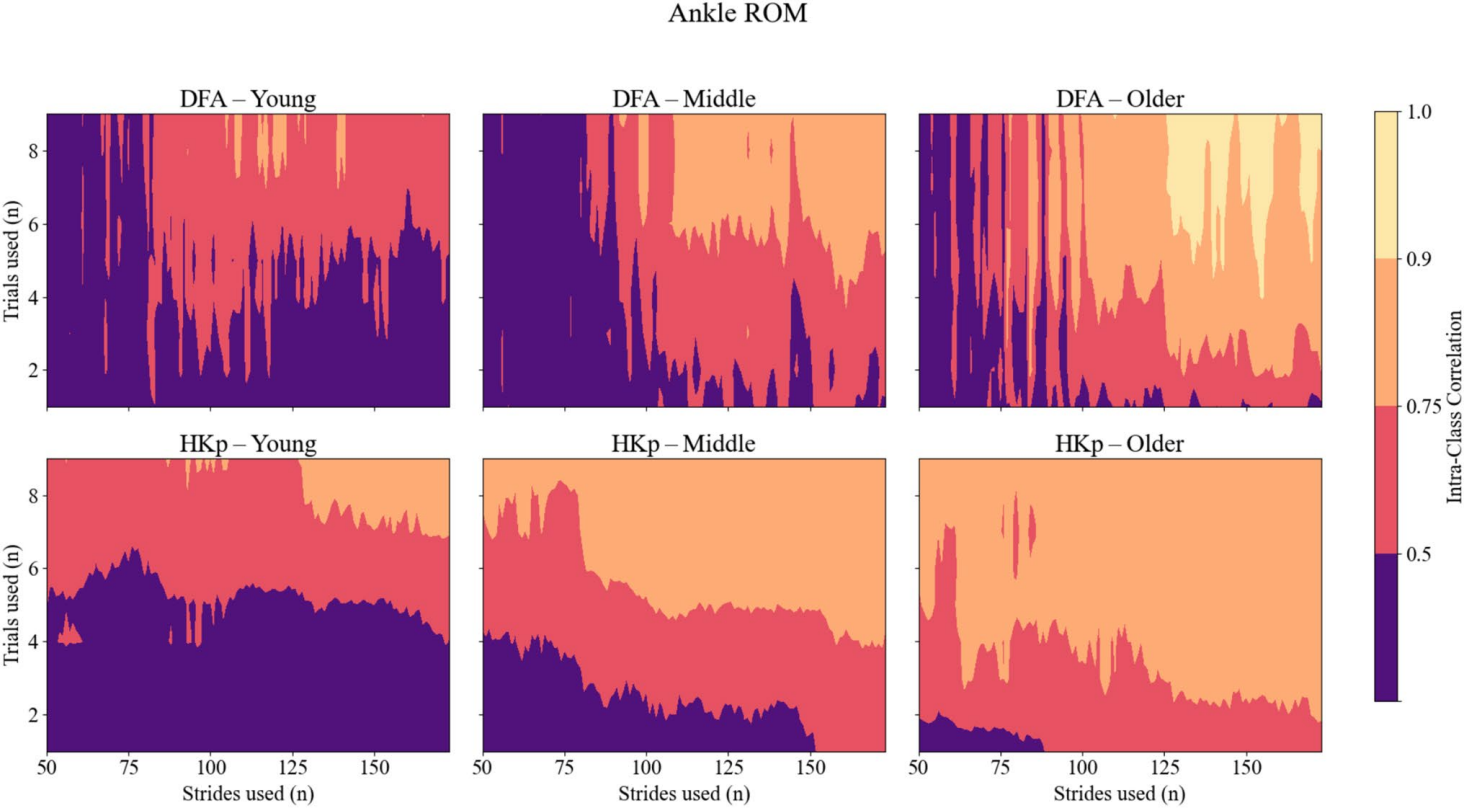
Intra-class correlation coefficients (ICCs) representing between-day reliability of *H* estimates for Ankle joint ROM time series across three age groups: young (left column), middle-aged (center column), and older adults (right column). Reliability is shown for two algorithms—Detrended Fluctuation Analysis (DFA; top row) and Hurst-Kolmogorov process (HKp; bottom row). The x-axis represents the number of strides per trial; the y-axis represents the number of trials used. Color gradients indicate reliability levels: purple (poor, ICC < 0.50), red (moderate, 0.50–0.75), orange (good, 0.75–0.90), and yellow (excellent, ICC > 0.90)

Similar patterns were observed for ICC of joint range of motion (ROM) *H*. Across all joints, DFA produced low to moderate reliability, with ICC values rarely exceeding 0.75. HKp demonstrated improved reliability across strides and trials used, particularly for the hip and ankle. While ICC values for ROM were generally lower than those observed for stride interval, HKp still achieved good reliability (ICC > 0.75) in many conditions when at least three trials and moderate stride counts were used. Group-specific analyses of ROM variables revealed similar trends, with HKp consistently outperforming DFA across all age groups (Figures 4, 5 and 6). Corresponding 95% confidence intervals for the variables presented in Figures 2, 3, 4, 5 and 6 are provided in the Supplementary Material (Supplementary Figures S1–S5).

### Simulated Data: Statistical Power Analysis

**Fig. 7:**
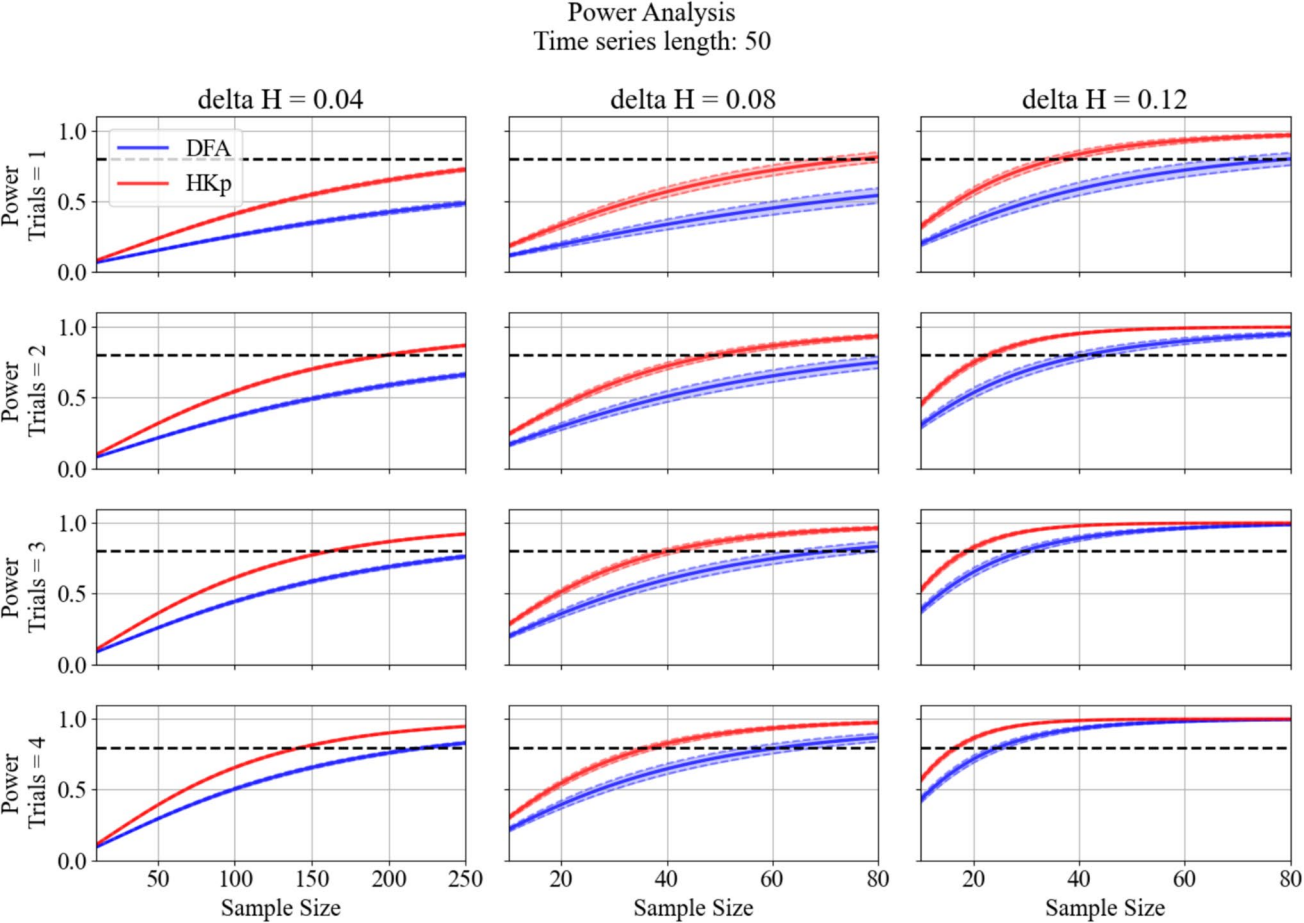
Statistical power estimates for detecting between-group differences in *H* using DFA (blue) and HKp (red), across a range of sample sizes and trial used. Columns represent increasing between-group mean differences (*ΔH* = 0.04, 0.08, 0.12), while rows represent the number of trials averaged per subject (1 to 4). Each curve reflects the average observed power for 1,000 iterations at α = 0.05. Shaded regions represent the confidence intervals of observed power across iterations. The horizontal dashed line denotes the conventional power threshold of 0.80. For *ΔH* = 0.04, simulations included up to 250 subjects per group to evaluate whether sufficient power could be achieved, whereas *ΔH* = 0.08 and 0.12 simulations included up to 80 subjects per group. The length of the simulated times series is 50 for all conditions

**Fig. 8:**
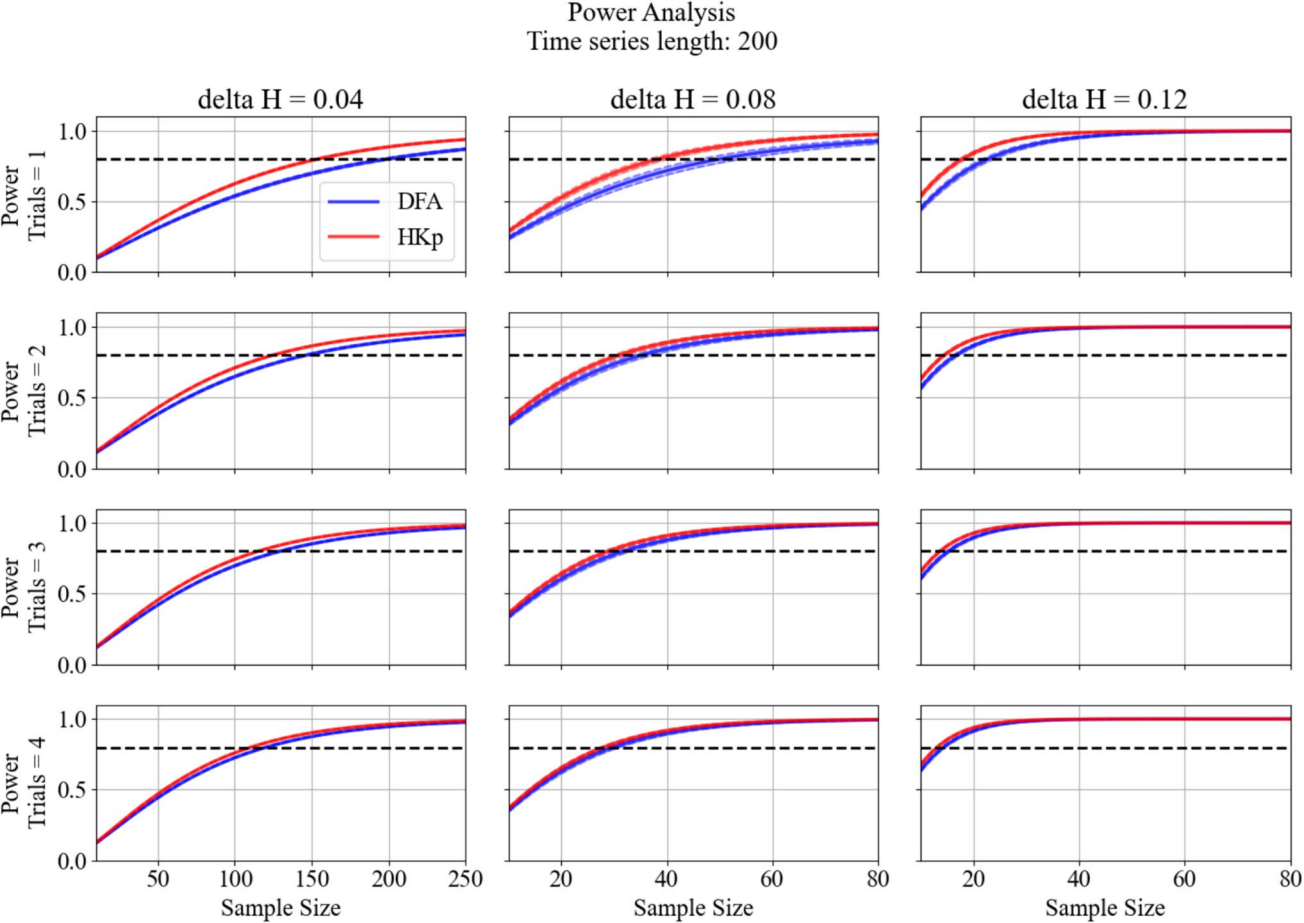
Statistical power estimates for detecting between-group differences in *H* using DFA (blue) and HKp (red), across a range of sample sizes and trial used. Columns represent increasing between-group mean differences (*ΔH* = 0.04, 0.08, 0.12), while rows represent the number of trials averaged per subject (1 to 4). Each curve reflects the average observed power for 1,000 iterations at α = 0.05. Shaded regions represent the confidence intervals of observed power across iterations. The horizontal dashed line denotes the conventional power threshold of 0.80. For *ΔH* = 0.04, simulations included up to 250 subjects per group to evaluate whether sufficient power could be achieved, whereas *ΔH* = 0.08 and 0.12 simulations included up to 80 subjects per group. The length of the simulated times series is 200 for all conditions

Simulated group comparisons revealed consistent power advantages for HKp over DFA across a range of effect sizes, sample sizes, and trials used. Figures 7 and 8 present the power for time series of 50 and 200 strides, respectively (see Supplementary Figures S6 and S7 for corresponding results at 100 and 150 strides). Across all timeseries lengths, HKp outperformed DFA, particularly when fewer trials or smaller group differences were used. At the smallest group difference tested (*ΔH* = 0.04), HKp required substantially fewer subjects to reach 80% power compared to DFA, which failed to reach the threshold even at 200 subjects in most conditions. At moderate (*ΔH* = 0.08) and large (*ΔH* = 0.12) effect sizes, HKp surpassed the conventional 0.80 power threshold with fewer subjects and trials compared to DFA. Increasing the number of trials per subject led to noticeable gains in power for both methods. These trends were consistent across all time-series lengths tested. Table 3 summarizes the minimum number of participants per group required to achieve 80% power across group differences (*ΔH* = 0.04, 0.08, 0.12), trials used (1–4), and time series lengths (50, 100, 150, and 200). Across all configurations, HKp consistently requires fewer participants than DFA, with the largest differences emerging with few trials used and small group differences.

**Table 3:** Minimum number of participants per group required to achieve 80% power

		50 strides		100 strides		150 strides		200 strides	
Trials used	*ΔH*	HKp	DFA	HKp	DFA	HKp	DFA	HKp	DFA
	0.04	> 250	> 250	190	> 250	165	235	154	198
1	0.08	77	> 80	48	78	40	55	39	50
	0.12	36	80	22	32	20	27	18	23
	0.04	198	> 250	143	196	131	163	125	147
2	0.08	49	> 80	37	52	32	39	31	36
	0.12	23	42	17	22	16	19	15	17
	0.04	162	> 250	128	162	120	140	116	130
3	0.08	41	71	33	42	30	35	29	32
	0.12	19	30	16	19	15	17	14	16
	0.04	144	226	120	145	114	129	111	121
4	0.08	38	62	31	37	29	33	28	30
	0.12	17	25	15	17	14	15	14	15

Values are reported for three effect sizes (*ΔH* = 0.04, 0.08, 0.12), four trial counts (1–4), and two algorithms (DFA and HKp). Time series length varied across 50, 100, 150, and 200 strides per trial. Values denoted as “ > 250” or “ > 80” indicate that 80% power was not reached within the maximum simulated sample size

## Discussion

In this study, we assessed the reliability and statistical power of the HKp algorithm for estimating the *H* from short time series of gait parameters, proposing it as a potential alternative to the widely used DFA. Our main aim was to assess the between-day reliability of *H* for stride interval, stride length, and the range of motion of ankle, knee, and hip joints. We also aimed to determine whether HKp estimates would result in increased statistical power compared to DFA using simulated data. We hypothesized that HKp would produce more reliable estimates than DFA, with reliability increasing as a function of stride count and number of trials. We also expected HKp to yield more accurate estimates and greater statistical power than DFA, with improvements scaling with the number of trials and subjects per group. Our findings supported both hypotheses.

Our findings showed that HKp yields more reliable *H* estimates than DFA across all gait variables tested. This finding was most evident for stride interval time series, where HKp achieved excellent reliability with fewer strides and trials used. Specifically, three trials and 100 strides were sufficient to produce ICC values exceeding 0.90. In contrast, DFA rarely surpassed moderate reliability under the same conditions, consistent with previous reports highlighting its sensitivity to time series length and underlying signal properties [14, 19]. Interestingly, ICC values for middle-aged adults were lower compared to young and older adults. It is possible that this group exhibited greater day-to-day variability in gait behavior that is unrelated to algorithmic estimation error. Low reliability may reflect rapid physiological changes (e.g. menopause) and elevated psychological stress (e.g. demanding work schedules), which are more likely to occur during midlife, and may contribute to greater day-to-day changes in *H*. Stride length generally exhibited lower reliability than stride interval, with HKp consistently outperforming DFA for this measure as well. This lack of reliability agrees with prior results that reported lower ICC values for *H* derived from stride length compared to stride interval [23]. While ROM variables yielded slightly lower ICCs overall, HKp still outperformed DFA, especially for the hip and ankle, indicating its robustness across both spatiotemporal and kinematic measures (Figures 3, 4 and 5). Importantly, this advantageous reliability was consistent across all age groups, suggesting that HKp provides stable performance regardless of participant’s age. However, ICC values derived from stride length and joint ROM systematically remained lower compared to stride interval ICC. This may reflect the heterogeneity of the joint ROM. Participants may achieve consistent stride interval *H* through multiple kinematic strategies, such that between-day variance in joint ROM *H* can coexist with more stable *H* in spatiotemporal variables across days. From a practical perspective, these findings suggest that HKp estimates of joint ROM should be interpreted with caution, especially when translating such metrics to clinical settings. At present, joint ROM *H* may be more appropriate for a scientific tool for studying gait variability across joints rather than as a standalone diagnostic marker. Taken together, these results provide evidence in favor of our first hypothesis, suggesting that HKp offers a more reliable method for investigating the temporal structure in gait variability, even for short walking trials.

Statistical simulations demonstrated that HKp offers greater statistical power than DFA, supporting our second hypothesis. This result was evident across all combinations of trial counts and sample sizes (Figures 7 and 8; see Supplementary Figures S6-S7). For the smallest group difference tested (*ΔH* = 0.04), DFA failed to surpass the conventional power threshold of 0.80 in all conditions, whereas HKp achieved sufficient power when larger sample sizes (up to 200 subjects) were used. This outcome was expected, given the parameters used for this simulation. The large sample sizes required for the smallest simulated effect (*Δ*H = 0.04) reflect the limited statistical power associated with very small true effect sizes, rather than limitations of the Hurst estimation method itself. At moderate (*ΔH* = 0.08) and large (*ΔH* = 0.12) effect sizes, however, HKp exceeded the 0.80 power threshold with fewer subjects and trials than DFA, highlighting its superior sensitivity to detect group-level differences. These findings expand on previous simulation work by testing a broader range of group differences [40]. Furthermore, whereas prior work demonstrated reduced statistical power in short time series with DFA, the present results show that higher power can be achieved with HKp [40]. Power curves for shorter (50 strides) and longer (200 strides) time series also demonstrated the same overall pattern. The steeper gains in power with increasing trial numbers suggest that HKp more effectively reduces within-group error variance, likely due to its capacity to extract accurate Hurst point estimates with minimal algorithmically induced error [13, 42]. Table 3 provides a practical summary for experimental planning, indicating the minimum number of participants required to reach 80% power across algorithms, trial counts, and effect sizes. Notably, DFA failed to reach this threshold in nearly all scenarios involving small to moderate effects, even with four trials per subject. In contrast, HKp achieved sufficient power with substantially fewer participants across all tested conditions. Collectively, these results indicate that HKp is not only more reliable but also more statistically efficient, making it superior for analyzing short walking trials. Nevertheless, these simulation results are based on configurations arising from our empirical dataset (e.g., within-group variability, between-trial reliability, and healthy adults) and should be interpreted as dataset-specific guidelines rather than plug-and-play sample size prescriptions. More broadly, this framework provides a template for evaluating method-specific reliability and statistical power before applying nonlinear analysis.

Our results have important implications for both clinical gait assessment and the broader use of nonlinear metrics in human movement science. The HKp method’s ability to produce reliable Hurst estimates from short time series makes it a promising tool for clinical use, where long walking trials may be impractical or fatiguing, especially for older adults and patients. For example, although standard guidelines recommend more than 512 strides, which typically requires at least 10 minutes of continuous walking [12], studies in Parkinson’s disease often apply DFA to shorter walking bouts lasting only 2 to 5 minutes [14, 22, 52, 53], which may be too short to draw valid conclusions about the temporal structure of gait variability. Replacing DFA with HKp can also have major consequences regarding experimental design and usage, like windowed analysis [54]. HKp reduces the burden on disadvantaged participants by requiring fewer strides and trials. This improvement also paves the way for the study of more demanding or unconventional tasks, such as blindfolded walking and load carriage, which require a limited trial duration. These advantages support HKp as a valid and practical alternative to DFA for assessing temporal structure in gait.

Despite the promising nature of our findings, we must acknowledge a few limitations. First, while our emphasis is on the clinical potential of HKp, we limited our reliability analysis to healthy individuals. As a result, the generalizability of these findings to clinical populations with impaired gait remains uncertain but an urgent matter. Second, while we evaluated a range of short time series lengths, we did not test longer durations exceeding 512 strides, where DFA is known to perform more consistently. For longer time series (> 512 strides), prior work has shown that DFA and HKp yield comparable Hurst estimates, as the impact of algorithmic bias diminishes with increasing data length [13]. Third, whereas DFA can be used on both stationary and nonstationary time series, HKp can only be used on stationary series, at least without additional preliminary processing. Fourth, both methods are biased by contamination of noise, strong linear and nonlinear trends, as well as short- and long-range cyclic trends [45]. Therefore, great care should be taken in the preliminary analysis of time series data to inspect for such anomalies using power spectral plotting, etc. Fifth, while not strictly a limitation, we must caution that the results we have presented apply to the types of simulations and data we have presented. Application of these methods to other sources of data would likewise require rigorous investigation to build confidence in other domains. We plan to address these limitations in future research. Finally, we made no effort to fine-tune DFA parameters such as detrending order, number of scales, and window lengths. Doing so may have produced different rules. That limitation, however, underscores a strength of DFA’s competitor. HKp requires fewer and less sensitive implementation choices compared to DFA, which may further strengthen its clinical applicability and reproducibility in research [43, 44]. In light of those limitations, we optimistically anticipate that other research teams will take up similar investigations in other contexts. Certainly, that is our intention in future research.

In summary, this study demonstrated that HKp provides more reliable and statistically powerful *H* estimates when compared to the traditional DFA algorithm in short walking trials. HKp achieved excellent reliability for stride intervals and improved performance across multiple kinematic variables, even with fewer strides and trials. These advancements support HKp as a promising tool for both experimental and clinical settings. By lowering the data requirements typically associated with nonlinear analysis, HKp expands the scope of studying gait in more challenging scenarios where 10-minute trials are not practical. To support future study design, we also provide a direct reference for the minimum number of participants (per group) needed to achieve sufficient statistical power across various experimental conditions (Table 3). For example, a researcher planning to find a 0.08 difference in *H* between groups with two trials containing 150 strides should collect data on no less than 32 participants per group. Overall, HKp can potentially offer a robust approach to calculate the Hurst exponent, particularly in short time series where DFA fails to reach good reliability and the conventional level of statistical power.

## Data Availability

The dataset analyzed in this study is publicly available as part of the NONAN GaitPrint databases: 10.1038/s41597-023-02704-z, 10.1038/s41597-024-04359-w, 10.1038/s41597-024-04359-w
